# Treatment of Dysentery

**Published:** 1852-11

**Authors:** 


*Treatment of Dysentery.* — The following paragraph expresses, in our opinion, much truth. We have acted upon it for years, and strongly commend it to the notice of our readers:

"But if the abstraction of blood be not the remedy for dysentery, I can speak with equal confidence, from my own experience, upon a point on which theory might be expected to be no less explicit—I mean the worthlessness of *mercury* for the subdual of the disease. At the time of which I have been speaking, I found my brother medical officers and the civil practitioners of the island, for the most part, agreed about the pretensions of mercury as a cure for the disease—pretensions which had had their rise in the extravagant opinions entertained of its merits among the Indian medical men. Thus, the rule that associated dysentery and the mineral in their practice, I

found to be "absolute" and unquestioned. My own experience, however, of its control over the disease, at first suggested doubts of its curative influence at all; and, latterly, convinced me that it was not merely inoperative, but prejudicial in the extreme. So far from the tenderness under pressure giving way, the tenesmus and tormina subsiding, and the blood and "corruption" composing the stools, giving place to normal dejections, under its use, the very opposite of all these was the case, in proportion as the poison was pushed and continued; and this so much as a rule, in my own practice, that I began at length to suspect the mineral as at the bottom of the mischief, and in the end, to be unable to disconnect the two. In order to avoid jumping to hasty conclusions upon the point in question, I administered the mineral in varied forms and doses—by the mouth and by inunction—in two grain doses and in twenty; and as a general rule, with the same results in both cases—to wit, an aggravation of the irritative action already going on in the mucous membrane—results which, as I have already said, I should have looked for from *priori* reasoning; and for these considerations:—1. That the state of the blood in dysentery is the opposite to that in which mercury is found by experience to be beneficial, being one of dyscrasy, and in which abnormal elements are superadded. 2. That I believe the natural tendency of mercury is to irritate all mucous membranes even in health, and greatly to aggravate irritation and inflammation when already present in the same. Such I believe to be the explanation of the poverty of its influence over mucous inflammations in general—a position, I am well aware, in which I shall not be supported by the general voice of the profession; but of the soundness of which I, myself, entertain no doubt.

What I have had to offer in relation to bleeding, leeching, and mercury, in this disease, is not less appropriate to the use of blisters for the same; in a word, that they are eminently impotent, if not injurious, in their operation.

So much for the worthlessness of the three most potent and valued agents in healthy inflammation, when addressed to the disorder now under consideration; and if this alone be not a strong ground of suspicion, as to the specific character of the action giving rise to it, I know not what stronger to adduce.

But as I am not writing a *treatise* upon dysentery and its management, it would be foreign to my present object, to enter further into what its essence *does* consist in, or to discuss at greater length the question, what treatment *is* adapted to its cure?

I may briefly mention here in passing, that I believe *opium*, in one form or other, to offer a larger promise of success in every variety of dysentery, than all the other agents in common use for it put together, provided the system be not further depressed by abstractions of blood, and that attention, at the same time, be paid to the all-important consideration of supporting the patient's strength, instead of sinking it by evacuants. I have stumbled upon a remark in a respectable authority of the last century (Kirkland "on Child-bed Fevers,") very apposite to the present subject, as well as suggestive of the inference, that Dr. Kirkland, as a physician, was in advance of the age in which he wrote. It is this: "I believe there is no kind of fever which will not be moderated by its use (that of opium); for if, in inflammatory fevers, we join small doses of it with antiphlogistics, and in putrid fevers with antiseptics, we shall frequently prevent the disease having violent effects, and give the proper medicines a better opportunity of accomplishing the intention desired."

"Were the same cases (of dysentery) again to be placed under my care, I would not hesitate to give opium in doses of four or five grains, as it was the opium chiefly that seemed to arrest the progress of the inflammation."—Cheyne, Loc. Cit.—*Lon. Med. Times & Gaz., from Western Lancet.*

